# Case Report: Epidemiologically inferred reverse zoonosis and intra-household zoonotic re-exposure of *Mycobacterium tuberculosis* in a domestic dog: a one health alert and the diagnostic potential of hypercalcaemia

**DOI:** 10.3389/fvets.2026.1824722

**Published:** 2026-06-18

**Authors:** Elisa Mazzotta, Tommaso Furlanello, Alissa Mazzei, Antonio Barberio, Federico Martignago, Mery Campalto, Greta Foiani, Maria Luisa Moronato, Claudio Battistella, Elisa Santinon, Michela Sabia, Valentina Carta, Maria Beatrice Boniotti, Federico Di Marco, Andrea Maurizio Cabibbe, Daniela Maria Cirillo, Alda Natale

**Affiliations:** 1 Istituto Zooprofilattico Sperimentale delle Venezie, SCT3 Padova, Vicenza e Rovigo, Legnaro, Italy; 2 Veterinary Clinic and Laboratory San Marco, Viale dell’Industria, Veggiano, Italy; 3 Servizio di Igiene Urbana Veterinaria AULSS 6 Euganea, Padova, Italy; 4 Dipartimento Di Prevenzione U.O.C. Igiene E Sanità Pubblica AULSS 6 Euganea, Padova, Italy; 5 National Reference Centre for Bovine Tuberculosis (NRC-TB), Istituto Zooprofilattico Sperimentale della Lombardia ed Emilia Romagna “Bruno Ubertini”, Brescia, Italy; 6 Emerging Bacterial Pathogens Unit, IRCCS San Raffaele Scientific Institute, Milano, Italy

**Keywords:** dog, hypercalcaemia, *Mycobacterium tuberculosis*, one health, reverse zoonosis, zoonotic transmission

## Abstract

**Background:**

Canine tuberculosis caused by *Mycobacterium tuberculosis* is rare and often underdiagnosed. This case report aims to describe the clinical, pathological, and molecular features of a dog with systemic tuberculosis, highlighting its epidemiological relevance within a One Health framework. The specific objective is to characterise a confirmed case of systemic *M. tuberculosis* infection in a dog, emphasising its unique clinicopathological features, including hypercalcaemia, and documenting an inferred reverse zoonotic transmission with subsequent intra-household zoonotic re-exposure.

**Methods:**

A 4-year-old male American Staffordshire terrier presented with lethargy, acute vomiting, anorexia, weight loss, generalised muscle atrophy, ataxia, and a history of chronic cough, orthopaedic disease, and dermatopathy treated with corticosteroids. Clinical evaluation revealed hypercalcaemia, oxaluria, proteinuria, and chronic respiratory and dermatological signs. Cytology showed granulomatous inflammation with intracytoplasmic negatively staining bacilli, raising suspicion for mycobacterial infection. Due to clinical deterioration, euthanasia was performed. Gross and histopathology revealed disseminated granulomatous hepatitis, nephritis, prostatitis, splenitis, and pneumonia with extensive caseous necrosis and mineralization. Ziehl–Neelsen staining confirmed intralesional acid-fast bacilli. Molecular diagnostics (PCR/RFLP, cgMLST) identified *Mycobacterium tuberculosis* complex (MTBC), and whole-genome sequencing revealed drug-susceptible *M. tuberculosis* lineage 4.1.2.1 (Haarlem), unrelated to other local strains. Epidemiological investigation supported reverse zoonotic transmission from an infected owner, with possible secondary transmission to a cohabiting individual.

**Conclusion:**

This case provides a detailed description of systemic canine tuberculosis and highlights hypercalcaemia as a potentially useful clinicopathological clue in veterinary patients with systemic granulomatous disease due to *M. tuberculosis*. The evidence supports a reverse zoonotic transmission of *M. tuberculosis* from human to dog, followed by onward zoonotic transmission to a cohabiting person who developed latent infection. This case underscores the importance of integrated cross-species epidemiological surveillance and the need to strengthen diagnostic and tracing protocols for companion animals within a One Health perspective.

## 1 Introduction

Canine infection with *Mycobacterium tuberculosis* (MTB) is a rare but increasingly recognised condition with significant zoonotic implications. Transmission from humans to dogs has been documented in both domestic and stray populations, often in households or environments with active human tuberculosis (1–4). Clinical presentations in dogs are variable, ranging from enteric and intra-abdominal forms (1, 5, 6) to pulmonary (4, 7) and cutaneous manifestations (8). Recent reviews have highlighted the growing number of confirmed cases and the diagnostic challenges they pose (9–11). Dogs may serve as sentinels or silent reservoirs in high-risk settings, particularly in rural or livestock-associated environments (12). The zoonotic risk is underscored by reports of occupational exposure among veterinary personnel during necropsy procedures (13).

Several previous reports have described hypercalcaemia (total and/or ionised) associated with infectious or parasitic granulomatous diseases in dogs, including angiostrongylosis, idiopathic granulomatous lymphadenitis, blastomycosis, and schistosomiasis (e.g., infection with *Heterobilharzia Americana*) (14–21). In this report, we describe a case of *Mycobacterium tuberculosis* infection in an owned dog, in which we highlight hypercalcaemia as a potentially significant clinicopathological finding in dogs with systemic granulomatous disease caused by *M. tuberculosis*.

Whilst hypercalcaemia is sporadically reported in canine tuberculosis (9, 22), it is a recognised paraneoplastic and granulomatous marker in human patients with pulmonary and extrapulmonary TB (23–26). The underlying mechanism is thought to involve macrophage-mediated extra renal synthesis of calcitriol, leading to increased intestinal calcium absorption (27, 28). Cases of hypervitaminosis D and hypercalcaemic crisis have been described in people with MTB infection across age groups and organ systems (29–31).

To our knowledge, this case represents another sporadic report of hypercalcaemia associated with disseminated MTB infection in a dog, suggesting a potentially relevant diagnostic parameter for systemic granulomatous inflammation that warrants further investigation in veterinary medicine. It further highlights the importance of integrated surveillance and collaboration between veterinary and human medicine, reinforcing the One Health approach to infectious disease management across species.

## 2 Case history

A 4-year-old intact male American Staffordshire terrier, weighing 24.5 kg, presented to the Veterinary Clinic and Laboratory San Marco with a 5-day history of acute vomiting, anorexia, lethargy, and progressive weight loss. The dog was fed a commercial dry diet and lived both indoors and outdoors. Vaccination history was incomplete and not compliant with the vaccination guidelines (32). The dog had a history of chronic dermatitis, intermittent coughing, and a past allergic reaction. Previously, he underwent surgical repair for cranial cruciate ligament rupture. Multiple courses of corticosteroids (prednisolone) had been administered over time for dermatological and orthopaedic conditions.

## 3 Clinical findings

On clinical examination, the dog was depressed, ataxic, and showed generalised muscle atrophy. Mucous membranes were pale to sub-icteric. Neurological assessment revealed proprioceptive deficits in all four limbs. His body condition score was assessed to be 1/5, consistent with an emaciated state.

Initial diagnostic tests revealed moderate metabolic acidosis, moderate to severe anaemia, and thrombocytosis. A mild elevation in coagulation parameters was also observed. The serum sample was icteric and haemolytic. Protein electrophoresis reported an inflammatory pattern with α2 and *γ*-globulin peaks (Figure 1).

**Figure 1:**
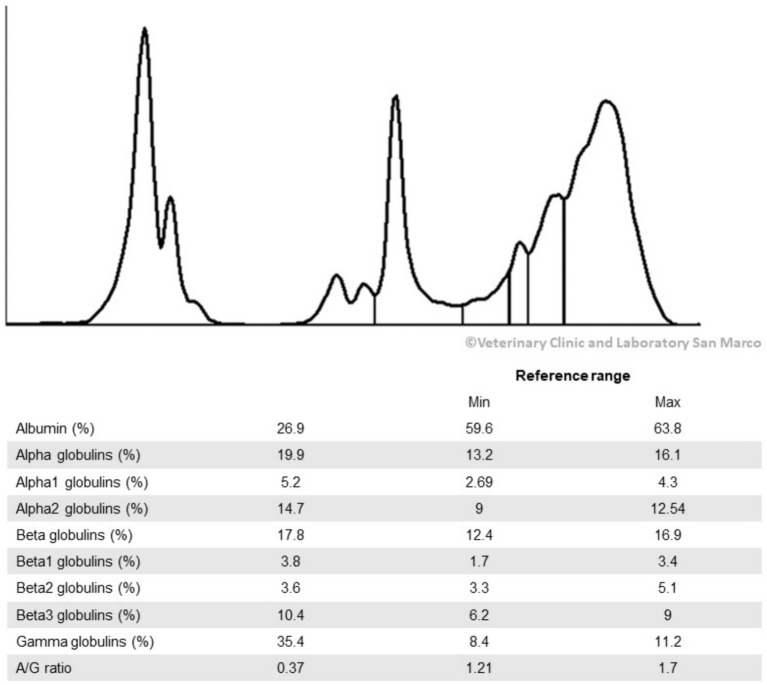
Serum protein electrophoresis profile of the dog. The densitometric trace reveals distinct peaks corresponding to the major serum protein fractions. The relative elevation of the gamma-globulin fraction in this profile suggests a polyclonal gammopathy, potentially linked to chronic antigenic stimulation. Reproduced with permission.

Biochemistry described a picture of severe inflammatory status along with kidney and liver failure. Notably, hypercalcaemia (12.9 mg/dL; reference range 9.8–10.9) and hyperphosphataemia (6.8 mg/dL; 2.8–3.9) were observed, with an increased calcium-phosphate product (Ca × *p* = 85.7 mg^2^/dL^2^; reference range 20–46.6), as shown in Table 1. Blood gas analysis confirmed the hypercalcaemia (1.68 mmol/L; reference range 1.29–1.40), while serum parathyroid hormone (PTH) was below the reference range (1.4–16.2 pg./mL).

**Table 1:** Selected laboratory findings (abnormal results only) for the dog at the time of admission.

Parameter	Result	Reference range
RBC	4.43 ×10^6^	7.1–8.22
Reticulocytes (/μL)	111,961	9,975–42,893
WBC	11.09 ×10^3^	6.52–11.67
Platelets	441 ×10^3^	157–329
CRP	2.66 mg/dL	0.01–0.03
Haptoglobin	524 mg/dL	33–92
Ferritin	612 ng/mL	59–168
CK	914 IU/L	59–164
AST	209 IU/L	22–41
ALT	183 IU/L	34–177
ALP	1,497 IU/L	16–38
GGT	10.9 IU/L	2.1–5.4
LDH	447 IU/L	10–110
Total bilirubin	3.52 mg/dL	0.13–0.26
Albumin	2.1 g/dL	3.1–3.7
Globulins	6.5 g/dL	2.8–3.4
A/G ratio	0.32	0.97–1.24
Triglycerides	136 mg/dL	25–65
Cholesterol	384 mg/dL	147–253
Urea	193 mg/dL	30–43
Creatinine	2.56 mg/dL	0.9–1.39
Calcium	12.9 mg/dL	9.8–10.9
Phosphorus	6.8 mg/dL	2.8–3.9
Ca × P product	85.7 mg/dL	20–46.6
aPTT	15.6 s	10.4–12.3
PT	9.9 s	7.4–8.35
Fibrinogen	303 mg/dL	149–223

RBC, Red Blood Cells; WBC, White Blood Cells; aPTT, Activated Partial Thromboplastin Time; PT, Prothrombin Time; CRP, C-Reactive Protein; CK, Creatine Kinase; AST, Aspartate Aminotransferase; ALT, Alanine Aminotransferase; ALP, Alkaline Phosphatase; GGT, Gamma-Glutamyl Transferase; LDH, Lactate Dehydrogenase; A/G ratio, Albumin/Globulin ratio.

Due to the complex hepatic and renal profile, additional serological investigations were performed to rule out potential infectious or parasitic comorbidities. Particularly, serological analysis using the microscopic agglutination test (MAT) for anti-*Leptospira* (*L*.) spp. antibodies revealed positive titres for *L. Grippotyphosa* var. Grippotyphosa (1:100) and *L. icterohaemorrhagiae* var. Copenhageni (1:800). Considering the diagnostic threshold of ≥1:100, as established by the World Organisation of Animal Health (WOAH) (2023) (33), the result indicates exposure to *Leptospira* spp. Quantitative ELISA for antibodies against *Leishmania infantum* was negative.

Hyposthenuria (SG 1.015), haematuria, proteinuria; PU/CU ratio 7.2 (ref: 0.1–0.3); calcium oxalate dihydrate crystals; urinary creatinine 19.6 (ref: 0.0–3.2) have been reported from the urinalysis.

Thoracic radiographs obtained in standard projections (right and left lateral recumbency and ventrodorsal view) revealed a diffuse nodular interstitial pulmonary pattern characterised by increased parenchymal opacity, reduced vascular definition, and bilateral reticulonodular infiltrates. Pleural thickening and mediastinal widening were also noted, suggestive of concurrent pleural effusion/lymphadenopathy.

Abdominal ultrasound (US) showed bilateral nephromegaly with parenchymal disorganisation, multiple confluent hypoechoic lesions, intralesional micromineralizations, and subcortical hypoechoic halo in both kidneys. Furthermore, hepatomegaly with diffusely hypoechoic parenchyma and millimetric intraparenchymal and ductal mineralisations have been described on liver US (Figures 2a,b).

**Figure 2:**
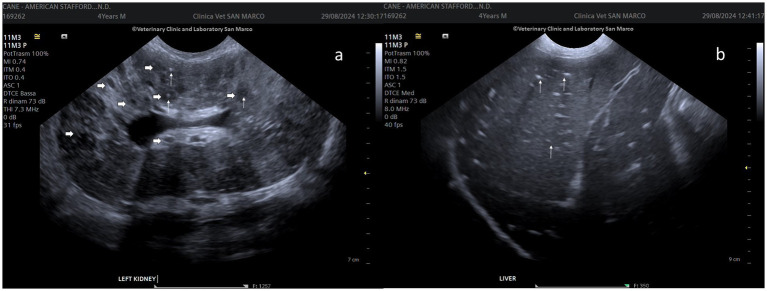
Abdominal US aspect of granulomatous lesions in *Mycobacterium tuberculosis* infection in an American Staffordshire terrier. **(a)** Left Kidney. Increased size, irregular profile with a mild hypoechoic sub capsular peripheral halo, diffuse increase in echogenicity with reduced/absent cortico-medullary distinction. Destructured parenchyma due to numerous disseminated roundish hypoechoic areas (⇒), confluent with each other and showing millimetric intralesional mineralisations (maximum diameter approximately 1 cm) (↑). **(b)** Liver. Diffuse decrease in echogenicity with fine-textured parenchymal echo structure, within which millimetric punctiform hyperechoic areas with posterior acoustic shadowing/reverberation are present (↑). Diffuse thickening and hyperechogenicity of the walls of the portal vessels.

Fine-needle aspiration (FNA) and cytological examination from the liver and kidney revealed macrophagic-neutrophilic inflammation with intracytoplasmic negatively staining rod-shaped structures within the macrophages (Figure 3).

**Figure 3:**
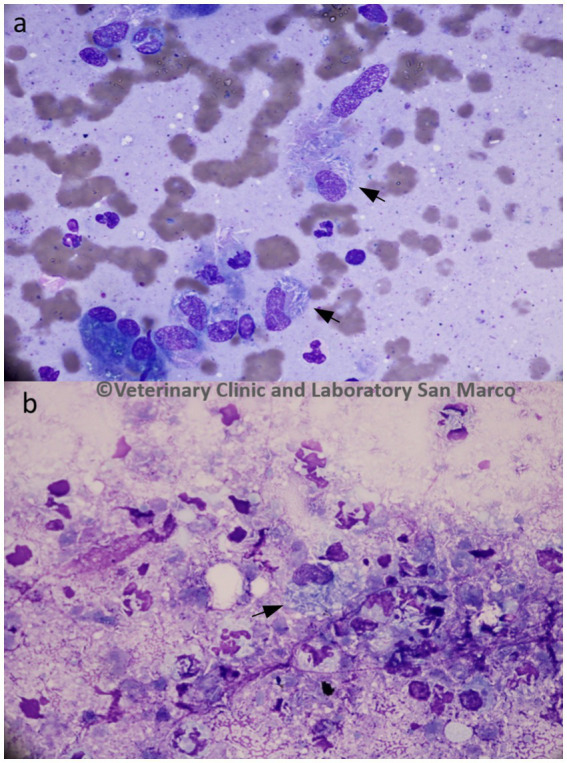
Liver and kidney fine-needle aspiration (FNA) cytology in an American Staffordshire Terrier with *Mycobacterium tuberculosis* infection. FNA samples from the liver **(a)** and kidney **(b)** show macrophages and bi- to multinucleated giant cells with abundant intracytoplasmic negatively staining rod-shaped structures (arrows) suggestive of *Mycobacteria* spp., and variable numbers of viable and degenerated neutrophils; background necrotic debris is evident in the kidney.

Despite intensive supportive care, the dog’s condition rapidly deteriorated, progressing to multi-organ dysfunction and showing additional abnormalities such as worsening azotaemia, marked electrolyte imbalances, and increasing hepatic enzyme activities. Euthanasia was elected due to the poor prognosis.

## 4 Gross pathology

At necropsy, generalised soft tissue pallor, mucous membrane sub-icterus, and skeletal muscle atrophy were observed. The skeletal musculature appeared diffusely pale and decreased in volume.

Mild bicavitary dark red effusions were present. The lungs were diffusely congested and oedematous, associated with a small amount of foam in the lumen of the deep airways. Multiple small, disseminated nodules were also visible throughout the pulmonary parenchyma.

The gastric mucosa exhibited multifocal necrotic and erosive lesions, with traces of digested blood within the gastric contents. The intestinal lumen throughout the tract contained catarrhal and dark, tarry material. The liver was enlarged, pale yellow with numerous miliary white nodules, exhibiting a firm, calcified texture upon sectioning. The spleen was enlarged and mildly pale. The kidneys were severely enlarged, irregular, and partially calcified upon sectioning. The renal parenchyma was markedly disrupted by multifocal to coalescing white-yellow nodules (up to 2.5 cm diameter), characterized by a greasy appearance and a caseonecrotic centre. The urinary bladder was dilated, with clear, bright yellow contents and scattered fibrin-like material. The prostate was enlarged, firm and calcified upon sectioning. The prostatic parenchyma was effaced by multifocal white-yellow caseonecrotic nodules (up to 0.5 cm in diameter), similar to those observed in the kidneys. Mild congestion of the penile portion of the urethra. The right testicle was retained within the abdominal cavity (cryptorchid) and markedly reduced in size. No macroscopic lesions were observed in the central nervous system. Representative samples of the lung, liver, spleen, kidney and prostate were collected and fixed in 10% neutral buffered formalin and processed routinely.

## 5 Histopathology

Representative samples of the lung, liver, spleen, kidney, prostate and brain were collected and fixed in 10% neutral buffered formalin and processed routinely. Histopathological examination revealed multiple granulomatous lesions affecting all examined organs, (Figure 4) with no lesions observed in the brain. These lesions were composed predominantly of epithelioid cells and fewer multinucleated Langhans-type giant cells, often surrounding central areas of necrosis. At the periphery, granulomas were variably circumscribed by lymphocytes, plasma cells, fibroplasia and fibrosis. In the liver, kidney and prostate, large caseous and colliquative necrotic areas were seen with variable areas of central mineralisation (Figures 4b,d), multifocally associated with deposits of mineralised coarse granular basophilic material. In the kidneys and prostate, necrotic areas were multifocally associated with low to abundant infiltrates of degenerated neutrophils (suppuration). Cytology of urine collected at necropsy revealed a mixed inflammatory population, predominantly composed of macrophages, degenerate neutrophils and fewer small lymphocytes. Rare intracytoplasmic negatively stained rod-shaped structures were observed in the cytoplasm of the macrophages (suggestive of *Mycobacteria* spp.). Numerous bilirubin crystals and fewer calcium oxalate crystals were also detected. The lungs and spleen were characterised by disseminated small granulomatous nodules (Figure 4f). Ziehl–Neelsen staining (Bio-Optica, Milan, Italy) demonstrated numerous acid-fast bacilli within the cytoplasm of epithelioid and multinucleated giant cells as well as dispersed throughout the necrotic debris (Figures 4c,e, inset). No relevant histopathological findings were detected in brain sections.

**Figure 4:**
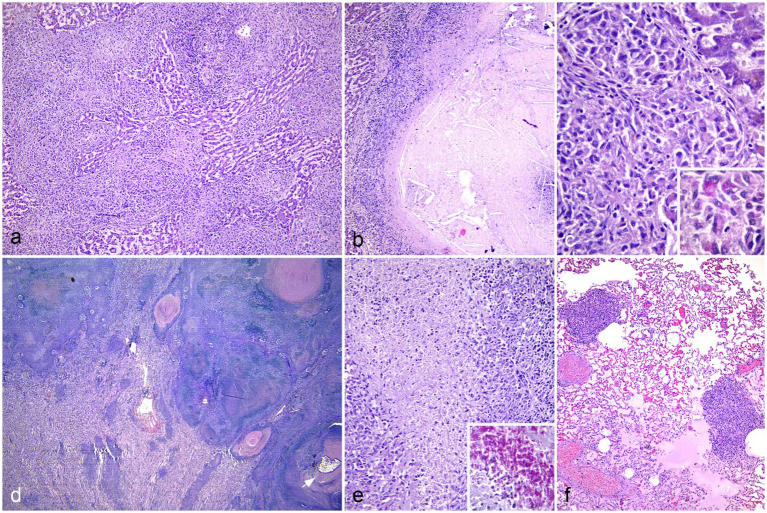
Histopathological findings in the liver, kidney, and lung of an American Staffordshire terrier with *Mycobacterium tuberculosis* infection. **(a)** Liver. Confluent, randomly distributed granulomatous nodules causing severe disruption of the lobular hepatic architecture. Haematoxylin and eosin (H&E). **(b)** Liver. Focal accumulation of caseous necrotic material containing cholesterol clefts and deposits of coarse granular basophilic material (mineralization), surrounded by lymphoplasmacytic infiltrates and fibrosis (H&E). **(c)** Liver. Epithelioid macrophages and bi- to multinucleated giant cells compressing hepatic laminae characterised by biliary canalicular stasis (H&E). Numerous intracytoplasmic acid-fast bacilli within macrophages and epithelioid cells (Inset, Ziehl–Neelsen staining). **(d)** Kidney. Extensive areas of caseous to colliquative necrosis, with granulomatous inflammation and multifocal suppuration, obscuring the renal cortex and partially extending to the medulla (H&E). **(e)** Kidney. Large area of colliquative necrosis, surrounded by granulomatous and lymphoplasmacytic infiltrates (H&E) and abundant extracellular acid-fast bacilli (Inset, Ziehl–Neelsen staining). **(f)** Disseminated small nodular granulomatous lesions scattered throughout the lung parenchyma (H&E).

## 6 Microbiological findings

Tissue samples from the dog were processed for both mycobacterial culture and molecular analysis by the National Reference Centre (CNR) for bovine tuberculosis in Italy, Istituto Zooprofilattico Sperimentale della Lombardia e dell’Emilia-Romagna (IZSLER). Briefly, a portion of each sample was degreased, shredded, and mechanically homogenised in physiological solution (1:2) using a stomacher for 2–5 min. The homogenate was divided into two aliquots, one subjected to decontamination for mycobacterial culture and the other used for direct molecular detection. For culture, the homogenate was decontaminated with 4% NaOH, neutralised, centrifuged, and the resulting pellet resuspended in phosphate buffer. Aliquots were inoculated onto Löwenstein–Jensen medium with pyruvate, Stonebrink medium, and liquid Middlebrook 7H9 MGIT, and incubated at 37 °C for up to 8 weeks.

In parallel, direct molecular detection performed on tissue homogenates by IS6110-based real-time PCR (34) revealed the presence of *Mycobacterium tuberculosis* complex (MTBC). Colonies compatible with MTBC growth were subsequently isolated in culture, and molecular identification confirmed the isolate as *Mycobacterium tuberculosis*. Following identification, bacterial colonies were suspended in Middlebrook 7H9 broth, heat-inactivated, sonicated, and subjected to DNA extraction using the NucleoSpin Tissue kit. DNA concentration was quantified fluorometrically. Whole genome sequencing libraries were prepared using the Illumina DNA Prep (M) Tagmentation kit and sequenced on MiniSeq platform, generating 2 × 150 base paired-end reads. Raw sequencing data, after quality and contamination check, respectively, with FastQC and Kraken, were subsequently sent to the IRCCS San Raffaele Scientific Institute and analysed using the MTBseq pipeline (35), with drug resistance interpretation based on the WHO mutation catalogue (36), and genetic relatedness assessed using Ridom Seqsphere+ software against a large local archive of WGS-characterised isolates, including those from Italy (37).

Genomic characterisation identified the isolate as *Mycobacterium tuberculosis* lineage 4.1.2.1 (Haarlem), with no resistance markers detected. Comparative analysis against national and international sequenced isolates revealed no genetically related strains based on core genome multilocus sequence typing (cgMLST) scheme at 5 and 12 allelic differences.

Further microbiological examination for aerobic microorganisms was performed on specimens and swabs collected during the *post-mortem* examination. Tissue aspirates were collected from lungs, liver and kidney and bladder contents with sterile swabs (Copan Italia S.p. A., Brescia, Italy).

Samples were then diluted in 1 mL of a nutrient broth (HIB, Heart Infusion Broth, Conda, Madrid, Spain), and 10 and 100 μL of bacterial suspensions were then inoculated into solid media and broths, respectively, as described below. The evaluation of aerobic microorganisms was conducted using a nutrient medium (BA, Blood Agar Base n°2, Biolife, Milan, Italy) with 5% defibrinated sheep blood (Allevamento Blood, Teramo, Italy), a nutrient broth (HIB), and selective Enterobacteriaceae media (McConkey agar, Oxoid, Basingstoke, UK). Cultures were inoculated and incubated at 37 ± 1 °C in aerobic conditions.

Bacterial culture yielded heavy growths of *Escherichia coli* (*E. coli*) and *Providencia stuartii* in the lungs with lesser growths of *Klebsiella* spp. *Pseudomonas* spp. was detected in the liver specimen. No other bacterial growth was observed in kidney and urine samples.

## 7 Epidemiological investigation: reverse zoonosis and zoonotic re-exposure

The epidemiological investigation conducted following the confirmed case of tuberculosis (TB) in the dog revealed that the animal was born and raised in the Veneto region and had never travelled abroad. The dog had been part of a stable household with two cohabiting owners for at least three years, both of whom were of Eastern European origin and frequently travelled to that region. One of the owners (A) had undergone treatment for severe active pulmonary TB in 2022. At that time, a contact investigation was initiated (January 2022), during which the second cohabitant (B) tested negative for TB infection (Quantiferon test and chest X-ray in February 2022; repeat Quantiferon in April 2022 also negative). At the time of the dog’s illness (September 2024), both owners (A and B) reported being asymptomatic (no cough, no fever). The dog was described as living both indoors and outdoors, frequently sharing space with a cat that appeared clinically healthy. The dog used to live in close contact with the owners. As part of the current investigation on October 2024, both owners underwent renewed TB screening (Quantiferon): the previously treated individual (A) showed no signs of active disease, while the second cohabitant (B) tested positive for latent TB infection and completed a preventive treatment regimen. In accordance with the protocol, owner B was re-evaluated in July 2025, with no clinical symptoms or abnormalities detected on routine examinations (Quantiferon negative). The results of the epidemiological investigation, including the documented infections within the household, are summarised in Figure 5.

**Figure 5:**
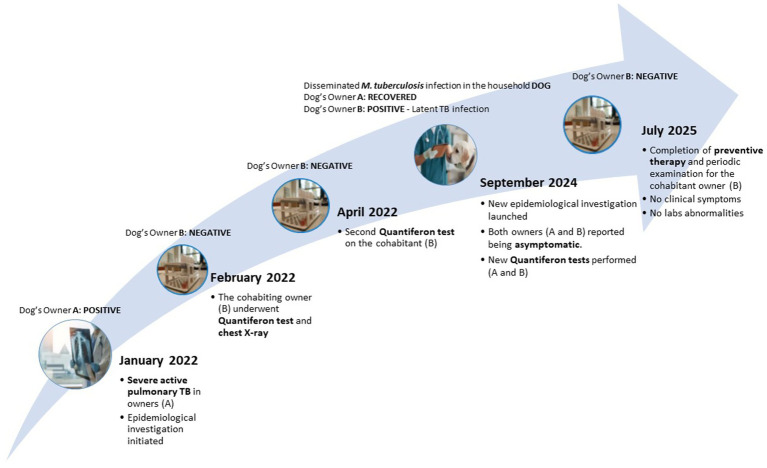
Timeline of the epidemiological investigation.

## 8 Discussion

This case describes a clinically under-recognised manifestation of canine tuberculosis and introduces hypercalcaemia as a potential useful biomarker in dogs with systemic granulomatous disease. It further reinforces the relevance of a One Health perspective, advocating for closer cooperation between veterinary and human medicine to improve detection and control of zoonotic diseases.

A particularly novel and clinically significant finding was the marked hypercalcaemia, accompanied by hyperphosphataemia and an elevated Ca × P product. In human medicine, hypercalcaemia is a recognised marker of active tuberculosis, attributed to extra renal synthesis of calcitriol by activated macrophages within granulomas (23–25, 27–29, 38–40). Recently, hypercalcaemia has been documented in cases of granulomatous steatitis, in which serum PTH, when assessed, was either undetectable or within the normal range, as observed in our case (14, 41). This finding supports the hypothesis of hypervitaminosis D as a secondary consequence of the underlying granulomatous condition (14). Indeed, this observation aligns with previous reports describing hypercalcaemia (total and/or ionised) in association with several infectious or parasitic granulomatous conditions in dogs (14, 16–21). To date, although this phenomenon has been documented in disseminated infections caused by various *Mycobacterium* species (9), it has only been rarely reported in dogs with confirmed *Mycobacterium tuberculosis* infection. This observation may therefore represent a rare veterinary documentation of such a metabolic disturbance associated with disseminate canine tuberculosis, highlighting a potentially clinically significant aspect of host-pathogen interaction in this species. The presence of granulomatous inflammation in multiple organs supports a similar pathogenic mechanism to that described in humans. This finding may offer a new diagnostic clue in dogs presenting with unexplained hypercalcaemia and systemic granulomatous disease.

The anatomopathological findings indicated a systemic mycobacterial infection, with the liver, kidneys, and prostate being most severely affected organs, and disseminated miliary foci detected in the lung and spleen. This pattern of distribution affecting the liver, lung, and spleen is consistent with a hematogenous (embolic) dissemination and raises questions about the route of infection. The animal may have acquired infection via inhalation, followed by early haematogenous dissemination to extrapulmonary organs. Alternatively, the predominance of lesions in the liver, coupled with comparatively limited pulmonary involvement, is compatible with a primary oral route of infection. Moreover, the extensive renal and prostatic lesions, along with the detection of rod-shaped structures within macrophages in urinary cytology, suggest the potential for urinary dissemination of the pathogen.

The prolonged administration of corticosteroids for the management of chronic disease may have predisposed the dog to increased susceptibility to opportunistic infections. The diagnosis of mycobacterial infection, and in particular that of infection with MTBC-group organisms, is notoriously challenging to confirm, as all the available diagnostic tests are limited by sensitivity, specificity, or both (9).

The diagnosis of *M. tuberculosis* infection in companion animals remains complex due to its nonspecific clinical presentation, and the rarity of confirmed cases and official reports.

The present case contributes to the growing body of evidence suggesting that dogs infected with *Mycobacterium tuberculosis* may serve as sentinel hosts for undiagnosed human infections within shared environments (42). Reverse zoonotic transmission, whereby humans transmit *M. tuberculosis* to companion animals, as inferred from this investigation, has been documented in several other reports, underscoring the need to include pets in contact tracing protocols, particularly in households with prolonged or close human–animal interaction (2, 43). Although environmental persistence of *M. tuberculosis* is considered limited compared to other mycobacterial species, its survival in aerosols and fomites may still pose a risk under specific conditions (44–46). In our case, the dog lived in close contact with its MTB–positive owner, a factor that significantly increases the likelihood of reverse zoonotic transmission. Dogs, due to their close physical proximity to humans and shared living spaces, may inhale infectious droplets or ingest contaminated biological material released during coughing or sneezing episodes by infected individuals. Unfortunately, we could not confirm the epidemiological link by genotyping of the two strains, as data from owner A was not available. Nevertheless, this transmission dynamic is supported by documented cases in the literature, where canine tuberculosis was traced back to human sources within the same household (2, 3, 47).

The strain isolate from the dog was submitted to the IZSLER (Italian National Reference Centre for Bovine Tuberculosis) and IRCCS San Raffaele Scientific Institute (TB Supranational Reference Laboratory) enabled definitive diagnostic confirmation and strain characterisation. Whole genome analysis identified the agent as *Mycobacterium tuberculosis* lineage 4.1.2.1 (Haarlem), a lineage widely distributed in Eastern Europe. Comparative genomic analysis against the reference archive of over 15.000 MTBC genomes, including national and international isolates, revealed absence of genetically related strains. The genetically closest isolates identified were two Haarlem strains from Kosovo; however, even these exhibited a distance of 38 alleles, a divergence incompatible with any recent transmission event. This substantial allelic gap, together with the fact that MTBC genomes-data collection of Eastern European isolates is not systematic, further supports the interpretation that the detected strain is unlikely to be autochthonous. These findings strongly suggest that the dog’s strain corresponds to an imported or extra-Italian genotype, rather than one circulating locally. This interpretation is fully compatible with the Eastern European origin of the dog’s owner and his frequent travels to that region.

Similar to other documented cases, diagnosis was achieved late in the disease course, which likely exacerbated clinical deterioration and delayed public health intervention (48–50). Human TB exposure was not initially included in the dog’s clinical history because the owner’s previous tuberculosis diagnosis was unknown at the time of presentation; this information was not disclosed to the veterinarian and is not routinely collected during standard canine anamnesis. This circumstance highlights the need to improve the completeness of clinical, anamnesis and epidemiological data collection in companion animals, particularly to strengthen surveillance and early recognition of severe zoonotic diseases. In addition, early diagnosis of TB in companion animals could reduce suffering, improve outcomes, and enhance epidemiological surveillance (47, 51, 52). The intimate nature of the human–animal bond in domestic settings underscores the importance of considering pets as potential secondary hosts, especially in regions or communities with high TB prevalence. Including animals in contact tracing and epidemiological assessments may enhance early detection and containment strategies within a One Health framework.

In addition, the novelty of this case lies in the potential secondary transmission from the infected dog to a cohabiting human, suggesting that reverse zoonosis may evolve into zoonotic re-exposure within the same household, a dynamic rarely documented in the literature (2, 47, 53).

This case report presents several strengths and limitations that warrant careful consideration. The confirmed diagnosis of *M. tuberculosis* and the comprehensive clinicopathological characterisation provide valuable insights into the presentation of systemic tuberculosis in a companion animal. Importantly, this case highlights the need to implement more structured and standardised diagnostic and epidemiological protocols for companion animals. While such protocols are well established in human medicine and are routinely applied in veterinary public health for livestock and within official control systems (e.g., food safety surveillance), they are not yet harmonised for pets. This case therefore underscores the relevance of extending coordinated diagnostic and tracing approaches to companion animals as part of a One Health framework.

The case also presents inherent limitations. As a single clinical observation, its generalizability is restricted. Moreover, the *M. tuberculosis* isolate from the human index case was not available, preventing direct strain comparison and limiting the completeness of the epidemiological investigation. This constraint hampers the ability to definitively reconstruct the transmission pathway and fully contextualise the infection dynamics.

Overall, the integration of veterinary and human clinical expertise, diagnostic workflows, and epidemiological investigation underscores the pivotal role of interdisciplinary collaboration in managing zoonotic diseases. This synergistic approach enhances surveillance capacity and supports the refinement of diagnostic protocols, enabling earlier detection and more accurate characterisation of emerging infectious threats at the human–animal interface.

## 9 Conclusion

This case highlights a rare presentation of disseminated *Mycobacterium tuberculosis* infection in a dog, with hypercalcaemia serving as a potential useful clinicopathological diagnostic clue in cases of systemic granulomatous disease in canines, including those caused by *Mycobacteria tuberculosis*. The integration of veterinary syndromic surveillance combined with human medicine epidemiological oversight underscores the importance of One Health collaboration, serving as a wake-up call for improved cross-sector vigilance. Notably, the epidemiological investigations enabled the inference of a reverse zoonosis event, with evidence suggesting human-to-animal transmission followed by latent infection in a cohabiting individual, indicating possible perpetuation of the pathogen within the household. This reinforces the need to enhance zoonotic monitoring and refine diagnostic protocols for early detection and control of emerging infectious diseases across species at the human–animal interface.

## Data Availability

The datasets presented in this study can be found in online repositories. The names of the repository/repositories and accession number(s) can be found at: https://www.ncbi.nlm.nih.gov/, PRJNA1433394.
